# P-1348. Impact of Minocycline Minimum Inhibitory Concentrations on Clinical Outcomes in Stenotrophomonas maltophilia Respiratory and Bloodstream Infections

**DOI:** 10.1093/ofid/ofaf695.1536

**Published:** 2026-01-11

**Authors:** Christine J Kubin, Nicholas Demenagas, Adrianna Losquadro

**Affiliations:** NewYork-Presbyterian Hospital, New York, New York; NYP/Columbia University Irving Medical Center, New York, New York; NewYork-Presbyterian Hospital, New York, New York

## Abstract

**Background:**

The Clinical & Laboratory Standards Institute (CLSI) recently lowered the minocycline breakpoint for *Stenotrophomonas maltophilia* infections from ≤ 4 to ≤ 1 mg/L due to results from one PK/PD study, however clinical data are lacking. This study assessed clinical outcomes between minocycline MICs (≤ 1 mg/L vs 2-4 mg/L) for *S. maltophilia* respiratory and bloodstream infections.

**Methods:**

This was a retrospective, cohort study of patients ≥ 18 years of age hospitalized at NYP/CUIMC who received ≥ 48 hrs of minocycline for *S. maltophilia* infection. Primary outcome was frequency of treatment failure. Secondary outcomes include isolation of *S. maltophilia* on follow-up cultures, development of minocycline resistance, and 30-day in-hospital mortality. Characteristics were compared between groups and between those with success/failure to construct a multivariable analysis for clinical failure considering all factors with a p-value < 0.2 and including MIC group.

**Results:**

80 patients were included: 63 with MIC of ≤ 1 and 17 with MIC of 2-4 mg/L. Majority (91%) were respiratory infections with 48 (60%) of patients being mechanically ventilated, 50 (63%) in the ICU at time of infection, and 35 (44%) were immunosuppressed. There was no difference in characteristics between groups and the Pitt Bacteremia Score was 3 in the MIC ≤ 1 group compared to 4 in the MIC 2-4 group (p=0.938). Treatment failure occurred in 41 (51%): 30 (48%) in the MIC ≤ 1 group and 11 (65%) in the MIC 2-4 group (p=0.328). Mechanical ventilation [30 (73%) vs. 18 (46%); p=0.025], ICU [31 (76%) vs. 19 (49%); p=0.024], and higher median PITT bacteremia score [5 vs. 2; p=0.001] were more common in those who failed compared to those with success. In the multivariable model, MIC 2-4 mg/L was not independently associated with treatment failure [OR 2.26 (95% CI 0.615-8.40; p=0.226).

**Conclusion:**

In patients who received minocycline for *S. maltophilia* respiratory and bloodstream infections, treatment failure did not differ between patients who had minocycline MIC of ≤ 1 mg/L compared to 2-4 mg/L. Although the results were not statistically significant, numerically there was a higher percentage of patients who failed treatment that had an MIC of 2-4 mg/L. More data with a larger sample size are needed.

**Disclosures:**

Nicholas Demenagas, PharmD, Ferring Pharmaceuticals: Advisory Board

